# Creativity and transition to bipolar disorder: a prospective analysis from the early-bipolife study

**DOI:** 10.1186/s40345-025-00406-6

**Published:** 2025-12-31

**Authors:** Elisabeth Michaelis, Michael Bauer, Andreas Bechdolf, Felix Bermpohl, Christina Berndt, Kyra L. Bröckel-Bundt, Eva Burkhardt, Christoph U. Correll, Udo Dannlowski, Irina Falkenberg, Andreas J. Fallgatter, Paolo Fusar-Poli, Sarina Hadji, Andreas Jansen, Georg Juckel, Tilo Kircher, Sarah Kittel-Schneider, Seza Krüger-Özgürdal, Martin Lambert, Karolina Leopold, Birgit Maicher, Silke Matura, Eva Mennigen, Pavol Mikolas, Andreas Reif, Philipp Ritter, Cathrin Sauer, Thomas Stamm, Julia Martini, Andrea Pfennig

**Affiliations:** 1 Department of Psychiatry and Psychotherapy, University Hospital, Faculty of Medicine, Carl Gustav Carus TUD Dresden University of Technology, Fetscherstrasse 74, 01307 Dresden, Germany; 2 Department of Psychiatry, Psychotherapy and Psychosomatics, Vivantes Hospital at Urban and Vivantes Hospital at Friedrichshain, Berlin, Germany; 3 Department of Psychiatry and Neurosciences, Charité Campus Mitte (CCM), Charité – Universitätsmedizin, corporate member of Freie Universität Berlin and Humboldt Universität zu Berlin, Berlin, Germany; 4 German Center for Mental Health (DZPG), Berlin-Potsdam site, Germany; 5 Psychiatric University Hospital Charité at St. Hedwig Hospital, Berlin, Germany; 6 Department of Child- and Adolescent Psychiatry, University Psychiatric Services, Bern, Switzerland; 7 Department of Child- and Adolescent Psychiatry, Charité Universitätsmedizin Berlin, Berlin, Germany; 8 Department of Psychiatry, The Zucker Hillside Hospital, Northwell Health, Glen Oaks, NY USA; 9 Department of Psychiatry and Molecular Medicine, Donald and Barbara Zucker School of Medicine at Hofstra/Northwell, Hempstead, NY USA; 10 Institute for Translational Psychiatry, University of Münster, Münster, Germany; 11 Department for Psychiatry and Psychotherapy, University of Marburg, Marburg, Germany; 12 Department of General Psychiatry and Psychotherapy, Tübingen Center for Mental Health (TüCMH), University Hospital Tübingen, Tübingen, Germany; 13 German Center for Mental Health (DZPG), partner site Tübingen, Germany; 14 Department of Psychosis Studies, EPIC Lab, King’s College London, London, UK; 15 Department of Brain and Behavioral Health Sciences, University of Pavia, Pavia, Italy; 16 Department of Psychiatry and Psychotherapy, LWL-University Hospital Bochum, Ruhr-University Bochum, Bochum, Germany; 17 Department of Psychiatry, Psychosomatic Medicine and Psychotherapy, University Medical Centre Frankfurt, Frankfurt am Main, Germany; 18 Department of Psychiatry, Psychotherapy and Psychosomatics, University Hospital Würzburg, Würzburg, Germany; 19 Department of Psychiatry and Neurobehavioural Science, University College Cork, Cork, Ireland; 20 Department of Psychiatry and Psychotherapy, University Medical Center Hamburg- Eppendorf, Hamburg, Germany; 21 Department of Psychiatry, Psychotherapy and Psychosomatic, Hospitals of Ruppin - Medical School Brandenburg Theodor Fontane, Neuruppin, Germany

**Keywords:** Bipolar disorders, Early detection, Creativity, Prevention, Risk factors, Prodrome, Predictors

## Abstract

**Background:**

Bipolar disorders (BD) are severe mental illnesses with recurrent depressive and (hypo-)manic episodes and a chronic course. While anecdotal and cross-sectional studies suggest a link between BD and creativity, longitudinal evidence is limited. This study investigates the role of creativity in individuals with varying risk for developing BD, using data from the multicenter, prospective Early-BipoLife study. *N* = 1,255 individuals aged 15–35 years were assessed and followed for over two years. Of these, *N* = 1,105 were included in the analyses; 150 were excluded due to missing creativity questionnaires. Creativity was measured with the Barron-Welsh Art Scale (BWAS) and the Creative Achievement Questionnaire (CAQ); BD risk was assessed with the EPI*bipolar*. Analyses included comparisons of mean creativity scores across BD risk groups and logistic regressions testing prospective associations between continuous creativity scores and transition to manifest BD. To enhance clinical applicability, group comparisons and odds ratios (ORs) were also calculated, providing estimates of relative risk across subgroups defined by BD risk status and creativity level.

**Results:**

At baseline (BL), participants at high BD risk scored significantly higher on the CAQ than those at low risk, while no differences were observed for BWAS scores. During FU, 25 of 1,105 individuals transitioned to manifest BD. Logistic regression analyses did not reveal significant associations between creativity and transitions. However, group comparisons indicated elevated transition likelihood in individuals with high BD risk, with the highest ORs in those combining high BD risk and high creativity (BWAS: OR = 7.05, 95% CI: 1.94–25.56; CAQ: OR = 5.57, 95% CI: 1.88–16.54) compared to low-risk individuals with low creativity.

**Conclusions:**

High BD risk was associated with higher CAQ scores at BL, suggesting heightened creativity may precede transition. Prospective analyses over two years did not confirm this association, likely due to the small number of transitions. Nonetheless, cross-sectional differences and group comparisons suggest that individuals with both high BD risk and high creativity, particularly real-world accomplishments captured by the CAQ, may show an increased likelihood of transition. These preliminary findings warrant replication in larger, longer-term studies. Importantly, creativity should not be pathologized but considered both as a resource and as a potential modifier of risk trajectories.

## Background

Bipolar disorders (BD) are severe mental conditions affecting about 1–3% of the population (Bauer and Pfennig 2005) and recent evidence from early detection centers has shown that help-seeking persons at risk for developing BD are often already affected by substantially impairing subsyndromal symptomatology years before the manifestation of BD (Leopold et al. 2012 ; Pfennig et al. 2020). Different risk factors were described and comprehensive risk assessment instruments such as the Early Phase Inventory of Bipolar Disorders (EPI*bipolar*, Leopold et al. 2012) were developed to comprehensively assess the proposed risk factors (e.g., positive family history for BD, mood swings, anxiety disorders, sleep disturbances (Leopold et al. 2012, 2020; Duffy et al. 2010; Bechdolf et al. 2010, 2012)).

In the 1980s, Andreasen and Glick (Andreasen and Glick 1988) proposed a potential link between creativity and BD, observing a disproportionately high prevalence of affective disorders among prominent artists. However, their conclusions were based primarily on anecdotal data. Subsequent studies examining more diverse and general populations have yielded mixed findings (Kaufman 2014). Some studies supported this link (e.g., Johnson et al. 2012; Rybakowski and Klonowska 2011; Richards et al. 1988; Kyaga et al. 2011), while others did not (e.g., Santosa et al. 2007; Simeonova et al. 2005). For example, Richards et al. (Richards et al. 1988) reported peak creativity in cyclothymic patients and first-degree relatives of BD patients compared to those with manifest BD, suggesting that personal functioning may moderate creative expression. Interestingly, a meta-analysis of 13 studies conducted by Forthmann et al. (Forthmann et al. 2023) found a small but significant association between divergent thinking and BD (d = 0.11). Divergent thinking refers to the ability to generate multiple novel solutions to open-ended problems. It is widely regarded as a key cognitive component of creativity and often used as a proxy for creative potential in psychological research. It emphasizes fluency, flexibility, and originality of ideas rather than finding a single correct answer (Torrance 1966; Guilford 1967).

Additional factors potentially associated with the relationship between BD and creativity include neurobiological mechanisms - such as the role of dopamine in reducing latent inhibition, a trait associated with creativity (Ashok et al. 2017; Flaherty 2005; Carson et al. 2003; Swerdlow et al. 2003) - as well as motivational processes, with ambition mediating the link between mania risk and creativity in a student sample (Ruiter and Johnson 2015). Finally, genetic evidence supports a shared basis: Power et al. (Power et al. 2015) showed that individuals with higher polygenic risk for BD were more likely to belong to artistic societies or pursue creative professions, suggesting that common genetic variants may simultaneously contribute to both heightened creativity and increased psychiatric vulnerability. This aligns with Carson’s Shared Vulnerability Model (Carson 2011), which proposes that overlapping cognitive and affective traits (e.g., cognitive disinhibition, attentional style driven by novelty salience, neural hyperconnectivity) underlie both creativity and psychopathology, with outcomes shaped by individual differences and moderating factors.

Given that pronounced creativity might already be present in individuals at risk for BD it might be relevant for the prediction and early detection of BD as well as for early preventive interventions (Burkhardt et al. 2019; Hadji 2021). However, only three studies investigated creativity in samples of individuals at risk for developing BD thus far. Burkhardt and colleagues (Burkhardt et al. 2019) assessed the risk status of 38 participants using early detection instruments (EPI*bipolar* and Bipolar-at-Risk (BAR)-Criteria, Bechdolf et al. 2012) and different creativity measures (Barron-Welsh Art Scale (BWAS, Welsh and Barron 1949), Creative Achievement Questionnaire (CAQ, Form et al. 2017; Carson et al. 2005). This study found that a higher risk score, defined by the EPI*bipolar*, was associated with higher BWAS scores indicating more pronounced creativity. Baseline (BL) data analyses of a subsample of our Early-BipoLife study also point out that high scores in creativity measures explained a third of the variance of the Bipolar Prodrome Symptom Scale (BPSS-FP, Correll et al. 2014a) score at BL after taking age and present hypomanic symptoms into account. Moreover, individuals with a high BD risk defined by the BAR-criteria had significantly higher mean scores in the CAQ than those with low risk (unpublished data, available on request). Given that these three studies were cross-sectional analyses no conclusion could be made about prospective associations between creativity and the transition to BD in high-risk individuals.

The present work aims to address this lack of research, using longitudinal data from the Early-BipoLife study to examine the prospective relationship between pronounced creativity in individuals at risk for BD and the risk of transition to BD.

## Methods

### Sample

**Fig. 1:**
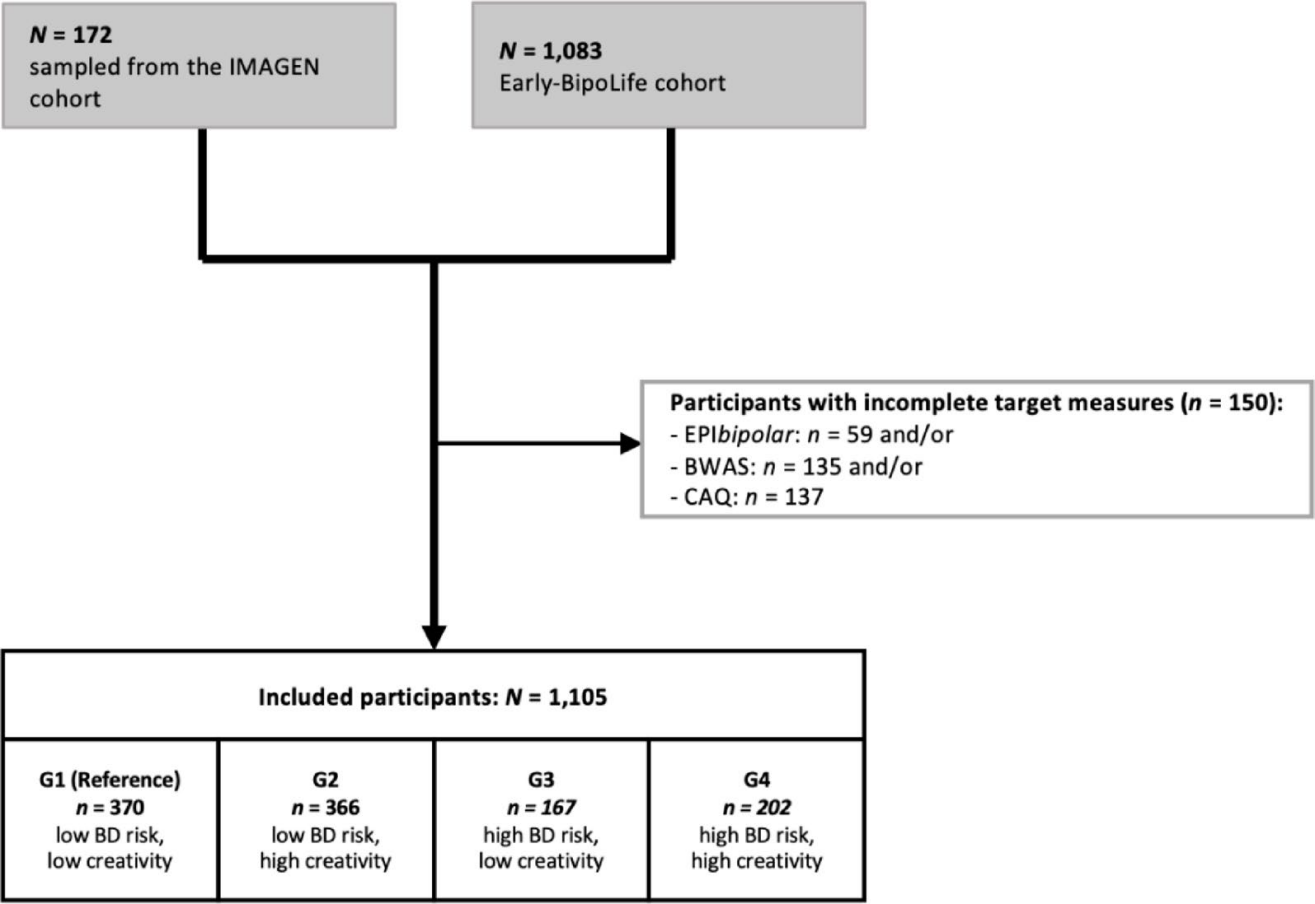
Participant flow chart of the analyzed sample (for further information see Martini et al., 2024). Risk status for bipolar disorders according to Early Phase Inventory for Bipolar Disorders (EPIbipolar, Leopold et al. 2012), creativity according to Barron-Welsh Art Scale (BWAS, Barron and Welsh 1949) and Creative Achievement Questionnaire (CAQ, Form et al. 2017; Carson et al. 2005), G1: group 1, G2: group 2, G3: group 3, G4: group 4.

The data were obtained from the Early-BipoLife study, a multicenter, prospective-longitudinal naturalistic cohort study with recruitment between 2015 and 2018. Help-seeking participants aged 15–35 years were assessed four times in the first two-year follow-up period (FU). Further participants with depressive syndrome or ADHD were recruited via in- and outpatient services. The study was conducted according to good clinical practice standards and has been approved by the responsible ethics committee of the Medical Faculty of the TU Dresden (No: EK290082014) and all local ethics committees of the other study centers in Berlin, Bochum, Frankfurt, Hamburg, Marburg, Neuruppin and Tübingen. Participants and in minors also their legal guardians provided written informed consent after comprehensive information about the study’s aims and procedures. The adolescents and young adults were included in the study when they were screened positive for at least one of the potential risk factors for BD (Pfennig et al. 2020). For the analysis, all participants with complete data in the early detection and creativity measures were included, thus, the final sample included *N* = 1,105 at-risk and control individuals (also see Fig. 1).

### Study design

For a detailed overview of the general study design see Martini et al. (Martini et al. 2024), Pfennig et al. (Pfennig et al. 2020), and Ritter et al. (Ritter et al. 2016). To examine whether creativity predicts the transition to BD in individuals at risk, we first tested the prospective association between creativity scores (BWAS, CAQ) and later transition to BD using a regression-based approach. This allowed us to assess whether higher creativity at BL was linked to an increased likelihood of developing BD over the FU period. In a second step, participants were divided into four groups based on their risk status according to EPI*bipolar* and their creativity levels at BL. This approach was chosen as in clinical practice often only the presence of “higher” creativity is registered even though this approach entails some loss of information. The reference group G1 (low BD risk, low creativity) was compared to G2 (low BD risk, high creativity), G3 (high BD risk, low creativity), and G4 (high BD risk, high creativity) with respect to their risk of transition to manifest BD. Risk classification followed the EPI*bipolar*, with participants categorized as “low BD risk” (no or low risk) or “high BD risk” (high or ultra-high risk). Creativity was classified as low or high using a median split of the BWAS and the CAQ (see Table 2). The main outcome was transition to manifest BD within the first two years of FU, as assessed by the Structured Clinical Interview for DSM-IV disorders (SCID).

### Procedure and measures

**Fig. 2:**
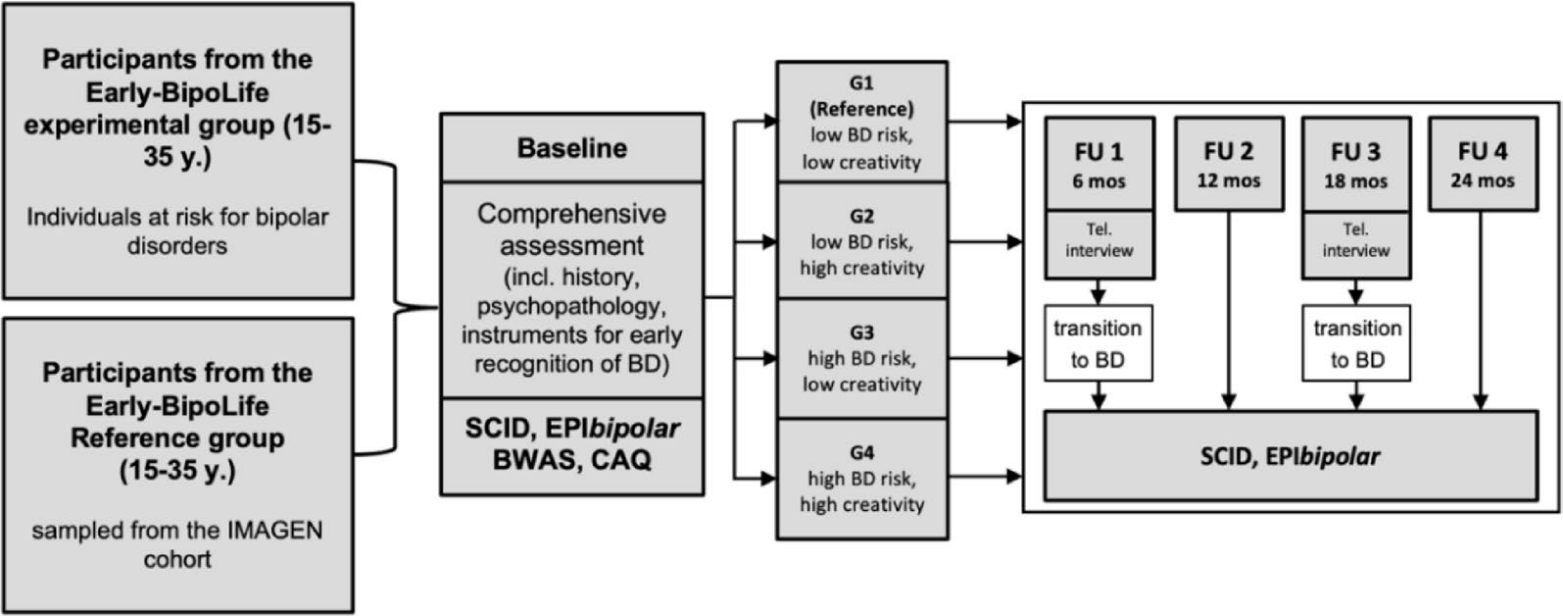
Study design of the Early-BipoLife study with important measures (for further information see Pfennig et al. 2020 ). SCID: Structured Clinical Interview for DSM-IV Disorders (Wittchen et al. 1997 ), EPI*bipolar*: Early Phase Inventory for Bipolar Disorders (Leopold et al. 2012 ), BWAS: Barron-Welsh Art Scale (Welsh and Barron 1949 ), CAQ: Creative Achievement Questionnaire (Form et al. 2017 ; Carson et al. 2005 ), G1: group 1, G2: group 2, G3: group 3, G4: group 4

During the BL assessment, sociodemographic and clinical characteristics of the participants were examined. A clinical evaluation followed, including semi-structured diagnostic interviews (SCID-I and SCID-II, Wittchen et al. 1997), early detection instruments (including the EPI*bipolar*) and a set of self-rating scales with two instruments assessing participant’s creativity (BWAS and CAQ, see below). During the FU assessments the clinical interviews were repeated focusing primarily on newly manifested psychiatric diagnoses (see Fig. 2).

Sociodemographic data and medical history were assessed conducting an extensive interview as part of the BL appointment.

The Structured Clinical Interview for DSM-IV Disorders (SCID-I/SCID-II, Wittchen et al. 1997) is a semi-structured clinical interview designed to assess psychiatric disorders according to DSM-IV criteria. The SCID has demonstrated good psychometric properties, with interrater reliability and test–retest reliability generally in the moderate to excellent range (κ ≈ 0.60–0.83), as well as good convergent validity with other standardized diagnostic instruments.

The EPI*bipolar* (Leopold et al. 2012) captures major (e.g., positive family history of BD, hypomania prodrome) and additional risk factors (e.g., impairment in psychosocial functioning, episodic course of symptoms) for BD. Besides the mentioned risk factors, it also takes information from the participant’s history, SCID diagnoses, and the mania risk of the BPSS-FP (Correll et al. 2014a) into consideration. Based on the information collected, a risk status is assigned, differentiating between no additional risk, low risk, high risk, and ultra-high risk.

The Barron-Welsh Art Scale (BWAS, Welsh and Barron 1949) is a nonverbal creativity test consisting of 86 black-and-white drawings with varying degrees of complexity, measuring creativity through a spontaneous aesthetic judgment. The BWAS is based on the assumption that creative persons prefer rather complex, asymmetric images as compared to more simple, symmetric ones (Welsh and Barron 1949; Barron 1953). Depending on the participant’s decision on each drawing, two scales are derived, the BWAS like scale (preference) and the BWAS dislike scale (rejection). In this study, the original rating of the BWAS was used, as opposed to the later definition of the revised BWAS. The original and the revised BWAS ratings are highly correlated, although the original BWAS scoring includes more items from the dislike scale (38 of 62 items, Welsh et al. 2005). According to a scoring key, each answer receives one or zero points, resulting in the BWAS score ranging from 0 to 62. With higher scores (i.e., a higher preference for complex, asymmetric images), higher creativity of the participant can be assumed. Even though the BWAS shows good psychometric properties in terms of reliability and validity (Welsh et al. 2005) it is not entirely clear whether the preference or rejection of the images is due to its complexity or other properties of the stimulus (Ridley 1977). Despite its limitations, the BWAS is regularly used for measuring creativity. It has been used in studies assessing manifest BD patients (e.g., Santosa et al. 2007; Nowakowska et al. 2005; Soeiro-de-Souza et al. 2011).

The Creative Achievement Questionnaire (CAQ, Form et al. 2017; Carson et al. 2005) is a self-report questionnaire measuring creative achievements across 13 domains (e.g., visual arts, music, creative writing) consisting of multiple parts. The first part assesses self-reported creative talent in these 13 domains. The second part lists eight questions for each domain that was ticked off in part one, asking for a ranking on a Likert scale from 0 (“no training or recognized talent in this area”) to 7 (e.g., “my work has been critiqued in national publications”). Since creative achievements depend on various factors that are only partially in control of the participant, two more parts were added. The third and fourth part capture creative interests and engagement, resulting in the CAQ achievements and CAQ interests subscores (Burkhardt et al. 2019). An overall CAQ score can be derived by adding these scores. The psychometric evaluation of the achievement parts established good predictive and convergent validity and internal consistency (Form et al. 2017; Carson et al. 2005). Similarly to the BWAS, there are studies that used the CAQ to investigate persons with manifest BD (Johnson et al. 2015; Vellante et al. 2011).

### Statistical methods

Statistical analyses were conducted using IBM SPSS Statistics V29 (Statistical Package for Social Sciences, version 29, IBM, Armonk, New York, USA). Participants with completely missing creativity data (i.e., no BWAS or CAQ scores) were excluded from the analyses. Multiple imputations were not applied, as imputing entire scales without any observed creativity data was deemed unreliable. Descriptive statistics were calculated to examine sociodemographic and clinical characteristics of the sample. Group comparisons were performed using Chi-squared tests (Chi²) and one-way analysis of variance (ANOVA), as well as independent-samples *t*-tests where appropriate, to examine whether the study groups differed significantly in their creativity scores as measured by the BWAS and CAQ. To investigate the potential impact of creativity in individuals at risk for BD on transition to BD, logistic regression analyses were conducted with creativity scores entered as continuous predictors to examine independent and interactive effects on transition to BD. In addition, odds ratios (ORs) and the corresponding 95% confidence intervals (CI) were calculated (G2, G3, G4 vs. G1 as well as G4 vs. G3), using both the BWAS and the CAQ as creativity measures.

## Results

### Distribution of creativity scores in the sample and the groups

**Fig. 3:**
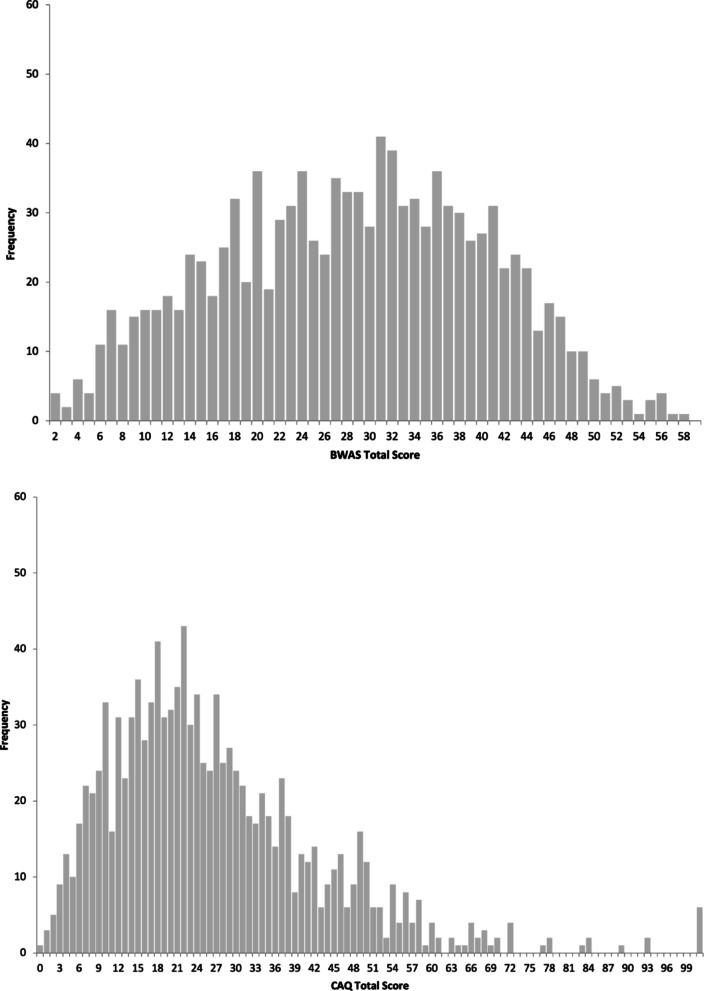
Distribution of creativity scores in persons at risk for bipolar disorders and controls (*N* = 1,105) measured with the Barron-Welsh Art Scale (BWAS) (**A**) and the Creative Achievement Questionnaire (CAQ) (**B**)

For the distribution of the BWAS and CAQ total scores see Fig. 3. BWAS total scores were normally distributed, whereas CAQ total scores were right-skewed as expected (Carson et al. 2005), in the present sample.

Table 1 shows descriptive statistics of BWAS and CAQ total scores in the EPI*bipolar* risk groups and in those with and without transition. Participants at high risk for BD scored significantly higher on the CAQ (M = 30.6, SD = 18.5) compared to those at no and low risk (M = 25.7, SD = 31.8, *t(1104) =* −2.74, *p =*.006). This finding was consistent when looking at the subscales of the CAQ, the CAQ achievements score (*p *=.070) and the CAQ interests score (*p* <.001, table on request). No significant differences emerged for BWAS total and subscores across risk groups (*t(1104) =* −1.41, *p* =.160).

**Table 1:** Comparison of mean creativity scores throughout the subgroups of the study sample (*N* = 1,105)

		Total	EPIbipolar risk		Transition	
			No and low risk	High risk	No	Yes
	***n***	1105	736	369	1080	25
**BWAS total**	**Mean (SD)**	28.5 (11.7)	28.1 (11.7)	29.2 (11.7)	28.4 (11.7)	32.2 (14.0)
	***t***		−1.407		−1.585	
	***p***		0.160		0.113	
**CAQ total**	**Mean (SD)**	27.34 (28.2)	25.7 (31.8)	30.6 (18.5)	27.1 (28.2)	36.1 (26.1)
	***t***		−2.738		−1.571	
	***p***		**0.006**		0.116	

BWAS: Barron-Welsh Art Scale, CAQ: CreativeAchievement Questionnaire

The assignment of the participants to the low and high creativity groups according to BWAS and CAQ based on the median split approach (with cut-off values of 28.5 and 27.3, respectively) is shown in Table 2. As can be seen, 44.3% of the participants were differently categorized with the two creativity measures. One third of the help-seeking at-risk participants did not show pronounced creativity combining the two instruments, one quarter, however, presented with high creativity scores in both.

**Table 2:** Categorization of the participants into the low and high creativity groups based on the Barron-Welsh Art Scale (BWAS) and Creative Achievement Questionnaire CAQ (*N* = 1,105)

		CAQ Score median split	
		Low creativity	High creativity
**BWAS Score median split**	Low creativity	357 (32.3%)	180 (16.3%)
	High creativity	309 (28.0%)	259 (23.4%)

BWAS: Barron-Welsh Art scale, CAQ: Creative Achievement Questionnaire

### Comparison of sociodemographic characteristics between the study groups

When using the CAQ to operationalize creativity, there were no differences regarding age, marital status, and current employment between the four study groups. Differences were found regarding sex (*p* =.032), education (*p* =.001) and occupation (*p* =.024, see Table 3). With the BWAS as creativity measure the differences regarding sex (*p* =.006) and occupation (*p* =.002) were also found, but no difference regarding education was detected (Table on request).

**Table 3:** Comparison of sociodemographic characteristics of persons at-risk for BD (*N* = 1,105) applying the CAQ for group assignment

		G1^a^ (*n*=370)		G2^b^ (*n*=366)		G3^c^ (*n*=167)		G4^d^ (*n*=202)		Group differences	*p*-value
		M	SD	M	SD	M	SD	M	SD	ANOVA	*p*
Age (years)		24.1	4.3	24.3	4.4	24.5	4.9	24.4	4.7	0.342	0.795
		***n***	**%**	***n***	**%**	***n***	**%**	***n***	**%**	**Chi** ^**2**^	***p*** ^**e**^
Sex (female)		240	50.4	151	58.1	113	59.5	115	64.2	**12.341**	**0.006**
Marital status	unmarried	454	95.4	242	93.1	179	95.2	162	90.5	-	0.255
(*n*=1,103)	married	17	3.6	15	5.8	6	3.2	12	6.7		
	divorced	5	1.1	3	1.2	3	1.6	5	2.8		
Education	attending school	15	3.2	13	5.0	9	4.8	12	6.7	-	**0.001**
(*n*=1,099)	other/no degree	5	1.1	1	0.4	2	1.1	2	1.1		
	middle school	135	28.5	28	10.8	62	33.0	31	17.3		
	high school	318	67.2	217	83.8	115	61.2	134	74.9		
Occupation	still in training	178	37.9	105	40.7	57	30.5	52	29.7	**25.818**	**0.002**
(*n*=1,090)	no professional qualification	120	25.5	72	27.9	59	31.6	60	34.4		
	qualification lower than college degree	115	24.5	33	12.8	42	22.5	37	21.1		
	college degree	57	12.1	48	18.6	29	15.5	26	14.9		
Current employment	yes	244	52.4	136	52.5	94	50.5	81	45.5	4.533	0.605
(*n*=1,089)	no	217	46.6	118	45.6	88	47.3	95	53.4		
	other (parental leave, gap year)	5	1.1	5	1.9	4	2.2	2	1.1		

*N*: number of participants, CAQ: Creative Achievement Questionnaire, *M*: Mean, *SD*: standard deviations, %: percentage of participants, Chi^2^: Pearson’s chi-squared test, ANOVA: analysis of variance, *p*: level of significance

^a^ G1: low BD risk, low CAQ; ^b^ G2: low BD risk, high CAQ; ^c^ G3: high BD risk, low CAQ; ^d^ G4: high BD risk, high CAQ

^e^Fisher’s Exact test was performed when more than 20% of the cells had an expected count of less than 5

### Risk for transition to manifest BD

To analyze the association of creativity measured by BWAS and CAQ with transition to manifest BD we conducted a logistic regression. Because of the significant differences regarding sex, education and occupation between the groups, they were included as confounders. Further, age was included as an additional potential confounder. The impact of creativity on transition to manifest BD was not significant (BWAS: B = 0.032; SE = 0.018; df = 1; 95% CI: 0.996–1.070; *p* =.083; CAQ: B = 0.004; SE = 0.003; df = 1; 95% CI: 0.998–1.010; *p* =.152).

**Fig. 4:**
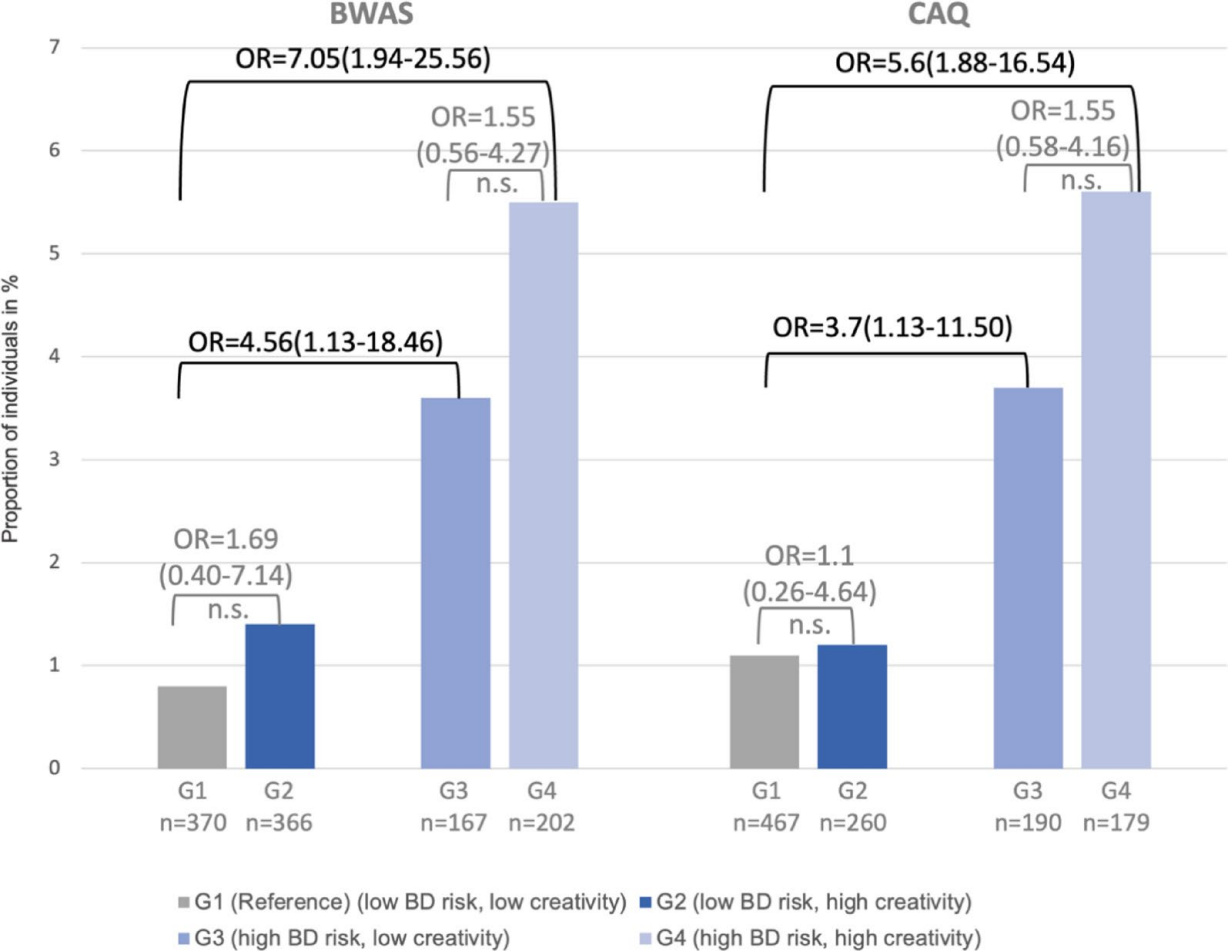
Comparison of transitions to bipolar disorders in four study groups with low or high risk for BD (EPI*bipolar*) and low or high creativity (BWAS and CAQ). BD: bipolar disorder, N: number of participants, BWAS: Barron-Welsh Art Scale, CAQ: Creative Achievement Questionnaire

To further explore the potential association between creativity and the risk of transition to manifest BD, we conducted group comparisons and calculated ORs, providing clinically interpretable estimates of relative risk across subgroups defined by BD risk status and creativity level. In the analysis with the BWAS creativity measure, 0.8% of the participants of the reference study group (G1, 3/370), 1.4% of group 2 (G2, 5/366), 3.6% of group 3 (G3, 6/167) and 5.4% of group 4 (G4, 11/202) transitioned to manifest BD within the first two years of observation (see Fig. 4). Compared to individuals with low BD risk and low BWAS creativity (G1, reference) no significant difference in the risk for transition to manifest BD was observed for individuals with low BD risk and high BWAS creativity (G2, OR = 1.69, 95% CI: 0.40–7.14, *p =*.503). Individuals with high BD risk and low BWAS creativity (G3) had an almost 5-fold higher risk for transition to BD (OR = 4.56, 95% CI: 1.13–18.46, *p* =.029), and individuals at high BD risk and high BWAS creativity (G4) had an even 7-fold higher risk for transition (OR = 7.05, 95% CI: 1.94–25.56, *p* =.001) compared to those of G1. No statistical difference was found comparing G3 and G4 (OR = 1.55, 95% CI: 0.56–4.27, *p* =.398).

Confirming results were observed when repeating the analyses using the CAQ to operationalize creativity: Individuals with high BD risk and low creativity (G3) had an almost 4-fold higher risk for developing manifest BD (OR = 3.60, 95% CI: 1.13–11.50, *p* =.045) whereas individuals at high BD risk and high CAQ creativity (G4) had an almost six-fold higher risk (OR = 5.57, 95% CI: 1.88–16.54, *p* =.001) as compared to the reference group G1 (see Fig. 4 ). Again, comparing G3 and G4 did not show a significant difference (OR = 1.55, 95% CI: 0.58–2.61, *p* =.581).

## Discussion

### Interpretation of the results

To our knowledge, this was the first analysis using longitudinal data to prospectively examine the role of creativity and the risk for transition from BD risk states to manifest BD.

The comparison of sociodemographic characteristics of the sample revealed significant differences between the study groups regarding sex, education, and occupation. Previous studies have shown that education might be related to creativity (e.g., Kapoula et al. 2016; Preiser et al. 2006), although these findings often stem from different populations such as children or students. Recent meta-analyses and large-scale assessments have generally reported small or inconsistent gender differences in creativity, with some evidence for a slight female advantage depending on context (Taylor et al. 2024; Abdulla Alabbasi et al. 2025; Goecke et al. 2026). The observed group differences in our study may therefore not necessarily reflect systematic sex-related effects on creativity but could rather result from sample-specific or contextual factors. These sociodemographic imbalances may reflect selection bias, whereby clinical or help-seeking samples differ in key characteristics compared to the general population. For example, individuals with psychiatric comorbidities are more likely to seek treatment, potentially skewing clinical cohorts toward certain demographic profiles (Tse et al. 2025). Thus, the observed differences may reflect indirect clustering of sex or occupational status within BD risk groups rather than a true association with creativity.

Cross-sectional BL analyses revealed that participants at high risk for BD scored significantly higher on the CAQ compared to those at no or low risk, whereas no significant difference emerged for the BWAS. This suggests, that heightened creativity - particularly in terms of concrete accomplishments and engagement in everyday creative activities - may already be present before the onset of manifest BD. The discrepancy between the two creativity measures likely reflects their different emphases: while the CAQ captures tangible creative achievements and active engagement, which may be more closely linked to personality traits and mood fluctuations that characterize BD risk states (Ruiter and Johnson 2015; Tse et al. 2025), the BWAS primarily assesses artistic preferences and aesthetic sensitivity, which may be less sensitive to clinical risk status. These findings underscore the importance of longitudinal FU to determine whether individuals with both high BD risk and high creativity scores are more likely to transition to manifest disorder.

Logistic regression analyses using continuous creativity scores (BWAS and CAQ) did not yield significant prospective associations with transition to BD. This discrepancy may be explained by several factors. First, the number of observed transitions within the two-year FU was small, limiting statistical power and reducing the likelihood that modest associations would reach significance. Second, cross-sectional comparisons relied on the full BL sample, providing greater power to detect mean differences across risk groups. Third, the CAQ assesses concrete creative accomplishments, which may be more closely aligned with clinical risk markers such as mood swings and increased activity levels, explaining why high-risk individuals scored higher on this measure but not on the BWAS, which captures self-perceived creative attitudes. Thus, CAQ differences may reflect co-occurring traits of individuals at heightened BD risk, without necessarily predicting short-term transition. Finally, creativity may impact risk only in interaction with other vulnerability factors, rather than exerting an independent main effect.

To further examine these relationships, we conducted categorical group comparisons and calculated ORs based on BD risk status and creativity levels. These analyses complemented the logistic regression models by offering a clinically interpretable perspective, highlighting a potential trend whereby individuals with both high BD risk and high creativity showed the greatest odds of transitioning to BD. This pattern was consistent across both creativity measures, which differ in their operationalization of the construct, indicating robustness across different assessments.

Although the significant differences in creativity scores in the risk groups at BL are suggestive, pronounced creativity alone was not associated with a statistically significant increase in transition risk, and the mechanisms underlying the potential link remain unclear. One possibility is that creativity reflects a cognitive-affective style that becomes maladaptive only under conditions of heightened mood dysregulation or reduced psychosocial functioning (Strong et al. 2007). Taken together, our results point to a complex relationship between creativity and BD risk: while creativity measures differentiated partially between levels of risk and showed mutual convergence across instruments, their predictive value for actual transition remains limited. These relationships should therefore be regarded as preliminary and call for replication in larger, prospective studies. Nevertheless, our findings suggest that creativity may represent a clinically relevant marker when considered alongside established risk factors, and could be cautiously integrated into early risk evaluations of help-seeking young people at elevated risk for BD.

### Strengths and limitations

The repeated and comprehensive assessments over two years are the major strengths of this work allowing for assessment of actual transition to manifest BD. However, the FU period of two years was comparatively short as other studies on BD transition risk report mean duration times of prodromal states between 1.8 and 7.3 years (Leopold et al. 2012; Correll et al. [Bibr CR16][Bibr CR34] ; Egeland et al. 2000; Faedda et al. 2014), which is longer than initially expected. With a longer FU period, more transitions to manifest BD will potentially be observed (Bechdolf et al. 2010, 2014). Another FU of the cohort is currently being conducted, which will allow to repeat the logistic regression analysis with potentially optimized statistical power.

There is no “gold standard” for measuring creativity (Thys et al. 2014a). We therefore used two established instruments - the BWAS and the CAQ - that have been applied in samples with manifest BD (Santosa et al. 2007; Vellante et al. 2011). Both measures, however, are limited. The BWAS primarily reflects preferences for complex visual stimuli and was originally developed to distinguish individuals with and without mental health problems (Barron and Welsh 1952; Eysenck 1983). It is therefore restricted to the artistic domain and may serve more as a proxy for aesthetic orientation than creativity itself. The CAQ, in turn, focuses on concrete achievements in recognized creative fields, which may bias results toward individuals with greater opportunities to pursue such activities. Moreover, creativity is a multidimensional construct. According to Kaufman and Beghetto’s Four C model (Kaufman and Beghetto 2009), it ranges from everyday expression (*little-c*) to professional (*Pro-c*) and eminent (*Big-C*) accomplishments, as well as personally meaningful insights (*mini-c*). Within this framework, the BWAS largely captures self-perceptions akin to *little-c*, while the CAQ emphasizes *Pro-c* achievements. This study therefore covers only a narrow segment of creativity, highlighting the need for broader measures in future research.

A potential limitation concerns the choice of the comparison and reference groups. Identifying an appropriate comparison group is challenging when studying creativity and affective disorders (Andreasen 2008). We compared high- and low-risk individuals. Creativity, however, may differ in other reference groups, including healthy controls, warranting further research.

Another limitation relates to the statistical methods applied. We additionally conducted logistic regression analyses using continuous creativity scores. These models, however, did not reveal significant associations with transition to BD, likely due to the small number of participants who transitioned. With larger sample sizes, it may be possible to detect significant differences where here only trends could be observed, providing more definitive evidence for potential interactions between creativity and BD risk in predicting transition. These advanced methods would allow to investigate associations with additional risk factors and potential mediators, such as mood fluctuations and temperament, which have been linked to increased creativity (Kyaga et al. 2011; Hadji 2021; Vellante et al. 2011). We chose to retain median-split group comparisons and corresponding ORs for interpretability and clinical relevance. Dichotomizing a continuous scale always comes with a loss of information. However, categorizing participants into “lower” and “higher” creativity groups enables clinicians to easier interpret and integrate this aspect into clinical reasoning than continuous scores.

### Clinical implications

The results suggest that creativity may interact with established risk factors to impact the likelihood of developing manifest BD. We were able to show that individuals with an increased risk of BD already exhibited higher levels of creativity. Participants at high risk for BD scored significantly higher on the CAQ compared to those at no or low risk, while no such differences were observed for the BWAS. This finding suggests that creative accomplishments and engagement in real-world activities (as captured by the CAQ) may be more closely aligned with clinical risk markers than self-perceptions or attitudes toward creativity (as captured by the BWAS). For this reason, the CAQ could be considered in the context of assessing young individuals at potential risk for BD, while also providing an overview of the patient’s creative interests and achievements to the clinician treating them.

In this prospective study we were able to observe individuals at risk for BD over a period of two years. Although logistic regression analyses did not yield statistically significant prospective associations, group comparisons revealed trends indicating a potentially higher transition risk in individuals with both high BD risk and high creativity.

Further, pronounced creativity may be detectable earlier than other clinical risk factors (Simeonova et al. 2005) and is often reported more readily, as it is perceived positively (Carson 2011; Hadji 2021; Johnson et al. 2016). Integrating creativity into comprehensive risk assessment tools could enhance early identification and provide additional context when differentiating between psychiatric disorders, as no clear association between high creativity and disorders such as schizophrenia has been observed yet (Thys et al. 2014b ). Creativity may also inform treatment planning. Knowledge of an individual’s creative potential could guide the selection of psychological or pharmacological interventions and improve adherence (Burkhardt et al. 2019; Murray and Johnson 2010). For example, individuals with high creative engagement might particularly benefit from complementary approaches such as art or music therapy alongside standard treatments (Carson 2011; Holm-Hadulla et al. 2021). Considering creativity as part of early intervention strategies may help reduce negative outcomes, including suicidality, comorbidity, and psychosocial burden (Leopold et al. 2020; Miklowitz et al. 2011; Pfennig et al. 2014).

## Conclusions

The association between creativity and BD has long been suggested, yet this area of research remains underrepresented. This cross-sectional and prospective analysis provides new insights into the role of creativity in individuals at risk for BD. At BL, participants at high BD risk scored significantly higher on the CAQ, suggesting that heightened creativity may already present before transition. While logistic regression analyses did not show prospective associations, group comparisons indicate a trend whereby individuals with both high BD risk and high creativity scores may be more likely to transition to manifest BD. These findings highlight the potential value of considering creativity in early detection assessments for help-seeking youth with elevated BD risk. Further research with larger samples and longer FU periods is needed to clarify the complex relationships between creativity, BD, and individual risk factors, and to determine whether the trends observed here can be confirmed with sufficient statistical power. Importantly, creativity should not be pathologized (Burkhardt et al. 2019); rather, it should be recognized as a resource that can inform both risk assessment and therapeutic planning.

## Data Availability

The datasets used during the study will be available from the corresponding author on reasonable request.

## Abbreviations

ANOVA: One-way analysis of variance
BAR-criteria: Bipolar-at-Risk-criteria
BD: Bipolar disorders
BL: Baseline
BPSS-FP: Bipolar Prodrome Symptom Scale
BWAS: Barron-Welsh Art Scale
CAQ: Creative Achievement Questionnaire
CI: Confidence Interval
Chi^2^: Chi-squared test
Early-BipoLife study: Improving early recognition and intervention in people at-risk for development of bipolar disorders study
e.g: Exempli gratia, for example
EPI*bipolar*: Early Phase Inventory for Bipolar Disorders
FU: Follow-Up
OR: Odds ratio
SCID: Structured Clinical Interview for DSM-IV Disorders
